# Genome-wide identification of microsatellite markers from cultivated peanut (*Arachis hypogaea* L.)

**DOI:** 10.1186/s12864-019-6148-5

**Published:** 2019-11-01

**Authors:** Qing Lu, Yanbin Hong, Shaoxiong Li, Hao Liu, Haifen Li, Jianan Zhang, Haofa Lan, Haiyan Liu, Xingyu Li, Shijie Wen, Guiyuan Zhou, Rajeev K. Varshney, Huifang Jiang, Xiaoping Chen, Xuanqiang Liang

**Affiliations:** 1 Crops Research Institute, Guangdong Academy of Agricultural Sciences, South China Peanut Sub-Center of National Center of Oilseed Crops Improvement, Guangdong Provincial Key Laboratory for Crop Genetic Improvement, Guangzhou, 510640 China; 2 MolBreeding Biotechnology Co., Ltd., Shijiazhuang, China; 3 Center of Excellence in Genomics & Systems Biology, International Crops Research Institute for the Semi-Arid Tropics (ICRISAT), Hyderabad, India; 4 Key Laboratory of Biology and Genetic Improvement of Oil Crops, Ministry of Agriculture, Oil Crops Research Institute of the Chinese Academy of Agricultural Sciences, Wuhan, 430062 China

**Keywords:** Genome sequence, Simple sequence repeats, Molecular breeding, Peanut (*Arachis hypogaea* L.)

## Abstract

**Background:**

Microsatellites, or simple sequence repeats (SSRs), represent important DNA variations that are widely distributed across the entire plant genome and can be used to develop SSR markers, which can then be used to conduct genetic analyses and molecular breeding. Cultivated peanut (*A. hypogaea* L.), an important oil crop worldwide, is an allotetraploid (AABB, 2n = 4× = 40) plant species. Because of its complex genome, genomic marker development has been very challenging. However, sequencing of cultivated peanut genome allowed us to develop genomic markers and construct a high-density physical map.

**Results:**

A total of 8,329,496 SSRs were identified, including 3,772,653, 4,414,961, and 141,882 SSRs that were distributed in subgenome A, B, and nine scaffolds, respectively. Based on the flanking sequences of the identified SSRs, a total of 973,984 newly developed SSR markers were developed in subgenome A (462,267), B (489,394), and nine scaffolds (22,323), with an average density of 392.45 markers per Mb. In silico PCR evaluation showed that an average of 88.32% of the SSR markers generated only one in silico-specific product in two tetraploid *A. hypogaea* varieties, Tifrunner and Shitouqi. A total of 39,599 common SSR markers were identified among the two *A. hypogaea* varieties and two progenitors, *A. duranensis* and *A. ipaensis*. Additionally, an amplification effectiveness of 44.15% was observed by real PCR validation. Moreover, a total of 1276 public SSR loci were integrated with the newly developed SSR markers. Finally, a previously known leaf spot quantitative trait locus (QTL), *qLLS_T13_A05_7*, was determined to be in a 1.448-Mb region on chromosome A05. In this region, a total of 819 newly developed SSR markers were located and 108 candidate genes were detected.

**Conclusions:**

The availability of these newly developed and public SSR markers both provide a large number of molecular markers that could potentially be used to enhance the process of trait genetic analyses and improve molecular breeding strategies for cultivated peanut.

## Background

Cultivated peanut or groundnut (*Arachis hypogaea* L.) is a globally important legume that is widely planted in Asia, Africa, America, and other areas because it is rich in seed oil and protein. Thus, peanut has great significance for fighting malnutrition and ensuring food security. In China, peanut accounts for almost half of the total output of all oil crops and is increasingly important as an oil and protein crop. Therefore, it is critical to improve peanut production and quality to ensure an edible oil supply.

However, peanut production is often constrained by factors such as drought, salinity, and disease [1]. During the past 10 years, with the development of peanut genomics, there have been several successful achievements in peanut trait mapping [2–5] and molecular breeding [6, 7]. Owing to the limited available genetic markers and low-density genetic maps, those studies could not provide optimal resolution of trait dissection and identify candidate genes. Therefore, the development of a high-density genetic map is particularly urgent for peanut trait mapping and breeding.

Simple sequence repeats (SSRs) are genomic fragments that consist of tandemly repeated units that are present in both coding and non-coding regions of the genome [8, 9]. SSR markers, designed by flanking sequences, are useful for and widely applied in plant genetic analyses and marker-assisted selection breeding. SSRs derived from expressed sequence tags (ESTs), transcriptome sequences, and genomic DNA sequences are referred to as EST-SSRs, transcriptome-SSRs, and g-SSRs, respectively. In the past decade, several hundred EST-SSR markers were developed by investigating ESTs [10, 11], and thousands of transcriptome-SSR markers were identified based on different transcriptome libraries of cultivated peanut [12–14]. Meanwhile, two integrated consensus genetic maps with thousands of different types of markers, such as EST-SSRs, transcriptome-SSRs, and g-SSRs, were constructed [15, 16]. Limited by the polymorphisms, these available SSRs were insufficient for constructing high-density genetic maps and enhancing molecular breeding [17].

Cultivated peanut is an allotetraploid (AABB, 2n = 4× = 40) that probably derived from hybridization between two diploids, *A. duranensis* and *A. ipaensis* [18, 19]. Recently, breakthroughs have been made in peanut genome sequencing. From 2016 to 2018, the genomes of the two diploid progenitors and one allotetraploid wild species, *A.monticola*, were successfully sequenced [20–23]. Based on the genomic sequences of the two diploid progenitors, genome-wide g-SSRs were identified and developed, and a high-density SSR physical map of wild peanut species was constructed [24]. Importantly, in 2019, cultivated peanut genomics research experienced a substantial milestone when genome sequencing was completed for three cultivated peanut : Fuhuasheng [25], Shitouqi [26], and Tifrunner [27]. The high-quality genome assemblies provide the opportunity for developing genome-wide g-SSR markers in cultivated peanut.

Here, we identified genome-wide g-SSRs and developed g-SSR markers from the genome assembly of *A. hypogaea* cv. Fuhuasheng, a landrace from North China that was sequenced in our previous work [25]. The aims of this study were to: (1) identify genome-wide SSRs and show the distribution of motif length, type, and repeat number between the two subgenomes (A and B); (2) develop g-SSR markers and construct a high-density SSR physical map of cultivated peanut; and (3) evaluate the application of these SSR markers and validate the polymorphisms in different cultivated peanut species. These novel, newly developed g-SSR markers could be helpful for advancing agronomic trait mapping, gene cloning, and molecular breeding of cultivated peanut in the future.

## Results and discussion

### Whole genome identification of SSRs

In this study, the whole genome sequence of *A. hypogaea* cv. Fuhuasheng was used to identify SSRs with different repeat motifs, from mono- to hexa-nucleotide. A total of 8,329,496 SSRs were obtained, with a density of ~ 3264.31 SSRs per Mb (Table 1; Additional file 1: Table S1). Penta-nucleotide was the most common type, accounting for more than half of all identified SSRs (57.75%), followed by hexa-nucleotide (26.61%) (Table 1). In addition, more SSRs were identified in subgenome B (4,414,961) than in subgenome A (3,772,653), and 141,882 SSRs were identified on the nine scaffolds of the peanut reference genome assembly (Table 1).

**Table 1:** Different types of SSRs identified in *A. hypogaea* L

SSR types	SSR Number	Proportion (%)	Subgenome A	Proportion (%)	Subgenome B	Proportion (%)	Scaffolds
Mono-nucleotide	255,774	3.07	112,466	2.98	138,624	3.14	
Di-nucleotide	376,173	4.52	167,574	4.44	202,132	4.58	
Tri-nucleotide	337,026	4.05	155,918	4.13	175,197	3.97	
Tetra-nucleotide	334,132	4.01	143,250	3.8	185,051	4.19	
Penta-nucleotide	4,810,032	57.75	2,189,218	58.03	2,538,767	57.5	
Hexa-nucleotide	2,216,359	26.61	1,004,227	26.62	1,175,190	26.62	
Total	8,329,496		3,772,653		4,414,961		141,882

Analysis of SSR distribution on each chromosome revealed that the largest number of SSRs was present on chromosome A07 (538,928), followed by chromosome B09 (532,326) (Additional file 1: Table S1; Additional file 2: Figure S1). Pearson correlation analysis revealed that chromosome length was significantly positively associated with the number of SSRs of each chromosome (*r* = 0.996, *p* < 0.01) (Additional file 1: Table S1). Furthermore, the average density of SSRs was 3283.89 SSRs per Mb, ranging from 3164.75 SSRs per Mb on chromosome A10 to 3843.36 SSRs per Mb on chromosome A08 (Additional file 1: Table S1; Additional file 2: Figure S1). Moreover, the densities of SSRs on chromosomes A08 and B03 were the highest in subgenomes A and B, respectively (Additional file 2: Figure S1).

A total of 501 types of SSR motifs were identified in the peanut genome (Additional file 1: Table S2). The repeat number of all of these motifs ranged from 2 to 335, most of which were concentrated in the top of 50s (Additional file 2: Figure S2a). The proportions of penta- and hexa-nucleotides with two repeats were much greater than those of other types in both subgenome A and B, and ranged from ~ 20 to 50% (Additional file 2: Figure S2b). Of the SSR motif types, the penta-nucleotide type, AAAAT/ATTTT, had the highest occurrence, and accounted for 9.55% of all types, followed by AAATT/AATTT and AAAAG/CTTTT, which accounted for 4.97 and 4.85%, respectively (Additional file 2: Figure S3). For each type of SSR motif, from mono- to hexa-nucleotide, the richest motifs were T (1.48%), TA (1.25%), AAT (0.37%), AAAT (0.34%), AAAAT (1.44%), and ACGCGT (0.64%).

### Genome-wide SSR marker development

The flanking sequences of all identified SSRs were used to design suitable forward and reverse primer pairs. A total of 973,984 SSR markers were successfully developed on 20 chromosomes and nine scaffolds in peanut (Additional file 1: Table S1), which accounted for 11.69% of all identified SSRs. Of these newly developed SSR markers, a total of 462,267 and 489,394 SSR markers were located in subgenomes A (Additional file 3: Table S3 and Additional file 4: Table S4) and B (Additional file 5: Table S5 and Additional file 6: Table S6), which accounted for 47.46 and 50.25% of all SSR markers, respectively (Additional file 1: Table S1). In addition, 22,323 SSR markers were mined on the nine scaffolds, and only accounted for 2.29% of all SSR markers (Additional file 7: Table S7).

**Fig. 1:**
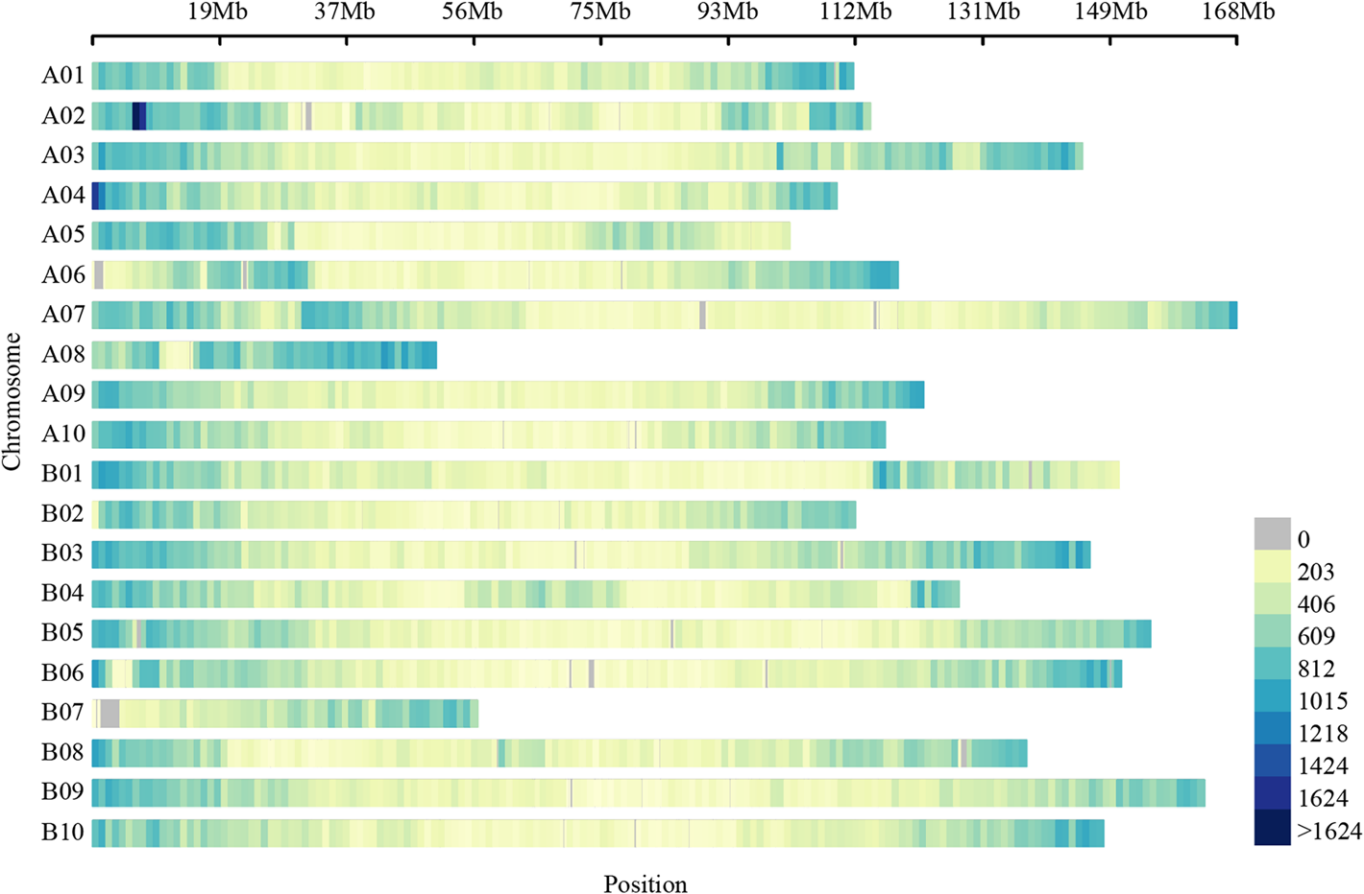
Overview of the high-density SSR physical map in peanut (*A. hypogaea* L.).The bar represents the number of SSR markers within a 1-Mb window

**Fig. 2:**
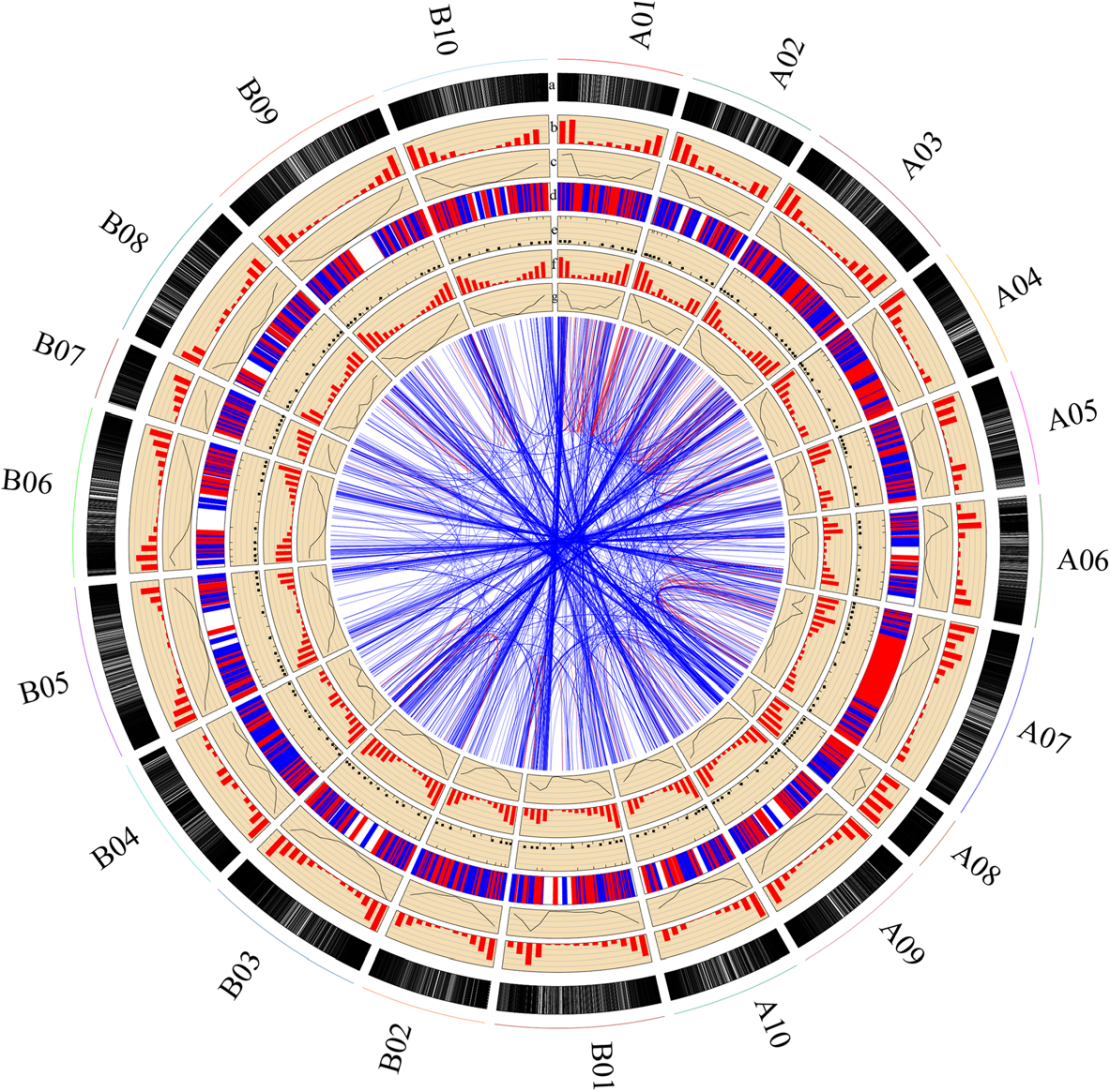
Genome-wide distribution of SSR markers on different chromosomes of the *A. hypogaea* genome. From the outer edge inward, circles represent the (**a**) gene position; (**b**) distribution of all genes; (**c**) gene density per Mb; (**d**) distribution of the 1276 integrated public SSR markers; (**e**) distribution of the 188 SSRs tested by PCR amplification; (**f**) distribution of all newly developed SSR markers; and (**g**) SSR marker density per Mb. Blue and red lines represent homologous loci in different and the same chromosomes, respectively

Based on the start positions of SSR markers, we successfully anchored these markers to the reference genome physical map (Fig. 1). For the physical map, the marker density significantly differed among chromosomes, and ranged from 338.38 per Mb on chromosome B01 to 699.92 per Mb on chromosome A08, with an average density of 392.45 per Mb (Additional file 1: Table S1; Fig. 2). Moreover, the distribution of each chromosome showed that there was lower marker density in the middle of each chromosome except for chromosome B07 (Figs. 1 and 2). In addition, for all SSR markers in subgenome A, subgenome B, and the scaffolds, penta-nucleotide SSRs were the most abundant (491,927) (Additional file 2: Figure S4a) and accounted for more than half of all SSRs (~ 50.51%) (Additional file 2: Figure S4b). Hexa-nucleotide SSRs were the second most abundant (199,408),and represented 20.47% of the SSRs in the two subgenomes and scaffolds, followed by compound and di-nucleotide SSRs. Of the repeat motif types, TA/AT was most abundant, accounting for more than 4.0% of all repeat motifs (Additional file 2: Figure S5). The second highest number of repeat motifs was AAAAT/TTTTA, which represented 3.4% of all repeat motifs. These analyses showed that the AT repeat patterns were the dominant repeat motifs of SSR markers, whereas GC repeat patterns were rare.

Development of new markers can help improve genetic analysis, gene/QTL mapping, and molecular breeding of crops. Currently, g-SSR markers are common and popular for such analyses, and they have wide applications in molecular genetics and breeding, because they have multiple advantages, such as simplicity, abundance, ubiquity, variation, co-dominance, and multi-allelism [28]. Recently, several studies were devoted to developing different types of SSR markers in peanut, such as EST-SSRs [11, 29], transcriptome-SSRs [12–14], and g-SSRs [30], even though the peanut genome had not yet been resolved. With the recent completion of genome sequencing of peanut and two diploid progenitor species, *A. duranensis* and *A. ipaensis*, a large number of genome-wide g-SSRs were identified [20–22]. Furthermore, tens of thousands of g-SSR markers (51,354 for *A. duranensis* and 60,893 for *A. ipaensis*) were also developed from the two progenitor species in 2017 [24]. However, there was limited reports on the development of a large number of genome-wide g-SSR markers from allotetraploid cultivated peanut because of the challenging in its genome sequencing. Fortunately, the genomes of allotetraploid *A. hypogaea* cv. Fuhuasheng, Shitouqi, and Tifrunner were successfully sequenced [25–27]. Here, large-scale genome-wide g-SSR markers were developed from the *A. hypogaea* cv. Fuhuasheng genome to help enhance genetic and genomic analyses and molecular breeding of peanut.

### *In silico* evaluation of the newly developed SSR markers

To evaluate the amplification specificity of the newly developed SSR markers, the forward and reverse primers of each SSR marker were used for in silico analysis based on the cultivated peanut genome sequences of *A. hypogaea* cv. Tifrunner and Shitouqi, and genome sequences of its two progenitors, *A. duranensis* and *A. ipaensis* (Table 2).

**Table 2:** In silico PCR products in *A. hypogaea* and its two progenitors

specise	0	1	2	3	4	5	≥6
*A. hypogaea* cv. Tifrunner	40,116 (4.10%)	855,422 (87.35%)	72,710 (7.42%)	7789 (0.80%)	1850 (0.19%)	600 (0.06%)	804 (0.08%)
*A. hypogaea* cv. Shitouqi	40,210 (4.11%)	874,384 (89.29%)	53,994 (5.51%)	7162 (0.73%)	1777 (0.18%)	667 (0.07%)	1097 (0.11%)
*A. duranensis*	581,706 (59.4%)	356,778 (36.43%)	34,490 (3.52%)	4668 (0.48%)	933 (0.10%)	293 (0.03%)	423 (0.04%)
*A. ipaensis*	532,523 (54.38%)	410,764 (41.59%)	30,814 (3.15%)	4120 (0.42%)	607 (0.06%)	199 (0.02%)	264 (0.03%)

The number of in silico products indicated that 87.35% (855,422) and 89.29% (874,384) of the SSR markers generated only one in silico-specific product in the twotetraploids *A. hypogaea* cv. Tifrunner and Shitouqi, respectively. Approximately 4% of markers were mismatched in the two cultivated varieties, and less than 8% of markers generated two products. In addition, less than 0.8% of SSR markers generated more than two products; in particular, only 0.08 and 0.11% of the markers generated ≥6 in silico products. For the two progenitors, a total of 356,778 and 410,764 SSR markers, which accounted for 36.43 and 41.59% of all SSR markers, respectively, generated only one in silico-specific product. However, more than half of total markers were mismatched in the two progenitors. Approximately 3% of all markers generated two in silico products, and less than 0.5% of the markers generated more than two in silico products (Table 2). In total, 1,729,806 and 767,542 markers could generate only one in silico product in cultivated peanut and its two progenitors, respectively. These SSR markers that only generated one in silico product would be potentially useful for molecular breeding in the future.

**Fig. 3:**
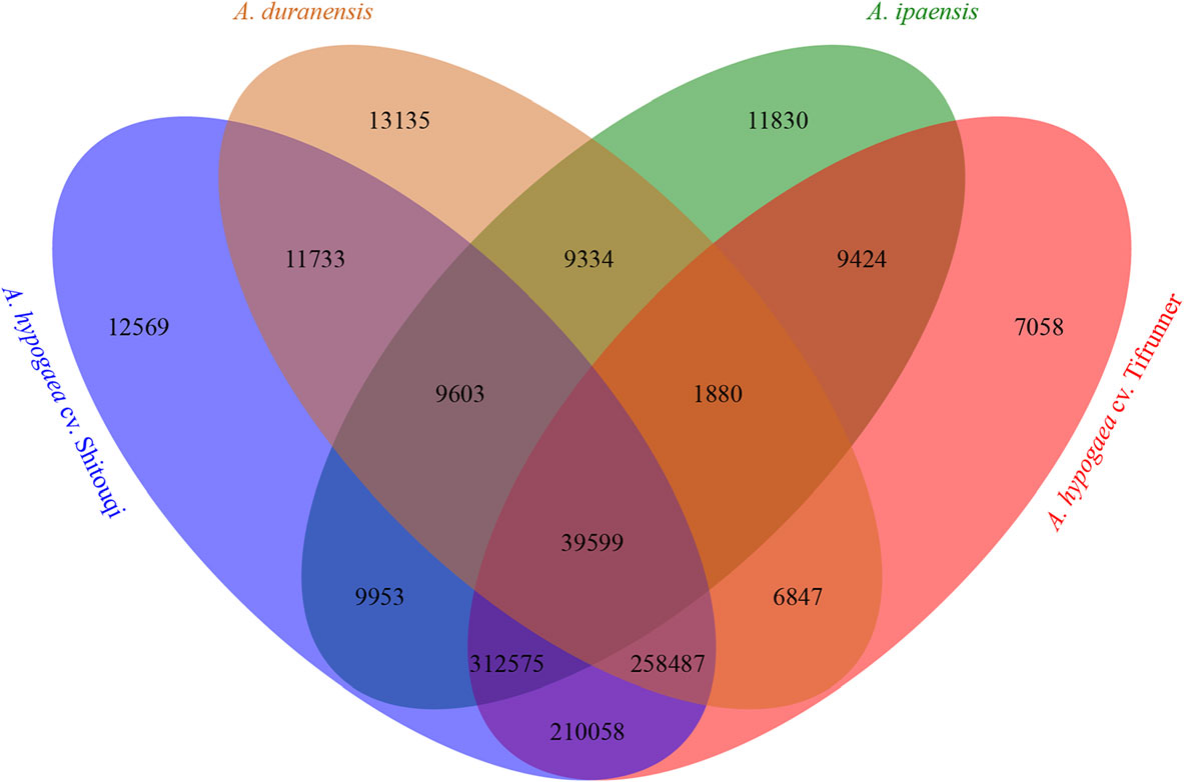
Venn diagram of SSR markers in different *Arachis* species

After clumped, a total of 39,599 SSR markers were shared among the four *Arachis* species, which indicates that these common markers were very conservative in different *Arachis* (Fig. 3; Additional file 7: Table S8). Moreover, 9334 and 210,058 markers overlapped between the two progenitors and two cultivated varieties, respectively. In particular, numerous specific SSR markers were also obtained for each genome, which indicates genomic polymorphism among the four different *Arachis* species.

### PCR validation of the newly developed SSR markers in different species

To validate amplification of the newly developed SSR markers, a total of 188 SSR markers with motifs ≥5 repeats were arbitrarily and evenly selected for PCR amplification in two cultivars, Fuhuasheng and Yueyouhei4hao (Additional file 7: Table S9). In total, 83 of the SSR markers (44.15%) could amplify clear target products in at least one of the two varieties (Additional file 2: Figure S6). Moreover, 25 of the 83 SSR markers displayed polymorphism between the two varieties. These results indicated that the newly developed SSR markers were available and could be useful for molecular breeding strategies, such as true hybrid F_1_ offspring selection, in the future.

### Integration of publicly available SSR markers

Previously, an integrated consensus genetic map, which contained 5874 markers, was successfully constructed with 20 linkage groups [16]. Based on forward and reverse primer sequences, a total of 5125 markers were mapped to the reference genome, *A. hypogaea* cv. Fuhuasheng. Finally, 3304 markers with 6838 loci were mined from 20 chromosomes (6699 loci) and nine scaffolds (139 loci) (Additional file 2: Figure S7). In addition, most of the markers (2927), which accounted for 88.6% of all markers, only matched one or two loci. For example, approximately 1832 markers only matched a single locus (Additional file 7: Table S10), and a total of 1095 markers were double loci identification on the reference genome (Additional file 2: Figure S7). The remaining markers had more than three loci, and one marker even matched 258 loci (Additional file 2: Figure S7). Furthermore, most of the multiple loci of these markers were located in the corresponding subgenomic chromosomes, such as A01 vs. B01 and A02 vs. B02 (Fig. 2). This may have been caused by co-evolution of the two peanut subgenomes.

Of these 1832 unique loci that matched markers, a total of 1276 SSR markers were located in the corresponding genetic linkage groups (Additional file 7: Table S10). Based on the physical positions of the 1276 publicly available SSR markers, they were successfully merged with the physical map and anchored to the draft genome sequence of tetraploid cultivated peanut (Fig. 2). This map, which has the highest marker density and the most uniform genetic background, will be of great benefit to molecular breeding, and gene and QTL mining of peanut in the future.

A high-density physical map with uniform genomic positions and coverage is necessary for conducting high-resolution gene/QTL mapping in crops. During the past years, several available QTLs for yield, disease resistance, and other traits were detected in peanut (https://www.peanutbase.org/). However, most of the QTLs were detected across different genetic backgrounds and environments, and only a few QTLs have been used for molecular breeding; therefore, the use of the QTLs in molecular breeding has been limited [31]. Thus, consensus genetic map construction was needed to improve the use of QTLs in molecular breeding. Therefore, two integrated consensus genetic maps with thousands of markers were constructed in 2013 and 2018 [15, 16], and multiple independent consensus QTLs were identified [16]. Here, to obtain a comprehensive consensus map, we integrated the published public SSR markers with the newly developed SSR physical map (Fig. 1). The availability of a high-density physical map would provide an opportunity to generate high-throughput genotyping data for different types of populations, and accelerate mapping and breeding applications of different traits.

### Application of SSR markers in peanut

High-density SSR physical maps can be used to screen more markers for QTL fine mapping. For example, a previous study reported that multiple novel QTLs for resistance to leaf spots and tomato spotted wilt virus were identified in peanut based on an improved genetic linkage map with a total of 418 markers, and multiple resistance QTL clusters were detected on linkage A05, which only contained 24 markers [32]. Based on the physical position of the marginal linkage markers (PM179–TC40D04), a total of 6804 new SSR markers were obtained in this region (Additional file 2: Figure S8a). On the A05 linkage, a total of 11 leaf spot QTLs were detected, especially for the QTL *qLLS_T13_A05_7*, which had the largest LOD value (5.26) and phenotypic variation explanation (15.55%) (Additional file 2: Figure S8b). Moreover, this QTL overlapped with our previous meta-QTL *MQTL_LLS_A05.1*, sharing the common marker GNB464 (Additional file 2: Figure S8b and c) [16]. Based on the sequences of the flanking markers near this QTL, GNB464 and TC40D04 were successfully anchored in a 1.448-Mb region on chromosome A05 in the *A. hypogaea* cv. Fuhuasheng reference genome (Additional file 2: Figure S8d). Searching this region in the high-density SSR physical map revealed a total of 819 newly developed markers (Additional file 2: Figure S8d). Gene identification of this region indicated that there were 108 genes, and functional annotation showed that multiple genes were related to disease resistance, such as the TMV resistance protein and NAC domain (Additional file 7: Table S11).

Genetic linkage mapping is an effective strategy for QTL identification in crops. However, a high-density genetic map is a prerequisite for QTL fine mapping of multiple phenotypic traits. In peanut, most QTLs were only preliminarily mapped because of the lack of sufficiently available genetic markers in the last decade [33–37]. Therefore, the development of molecular markers has become increasingly important. Then, high-density integrated consensus maps with thousands of markers were constructed in 2013 and 2018 [15, 16]. However, in cultivated peanut, development of high-density physical markers was rare. Here, we developed millions of SSR markers in cultivated peanut, and this large number of newly developed SSR markers, such as multiple QTLs on chromosome A05 or even on the region of *qLLS_T13_A05_7*, could provide more possibilities for further fine mapping or even gene cloning.

## Conclusions

Development of new genome-wide markers and construction of a physical map with uniformly distributed genome-wide physical markers would aid in the elucidation of complex traits and improvement of molecular breeding. In this study, we identified a total of 8,329,496 genomic SSRs and developed 973,984 newly genomic SSR markers from the cultivated peanut reference genome “Fuhuasheng” using MISA software with default parameters. Moreover, we mined a number of 39,599 common SSR markers from two *A. hypogaea* cv. Tifrunner and Shitouqi and two progenitors, *A. duranensis* and *A. ipaensis*. In addition, we integrated 1276 public SSR loci with the newly developed physical map, and obtained the first high-density genomic physical map for peanut. Finally, we fine mapped a leaf spot quantitative trait locus to a 1.448-Mb region by marker encryption and identified 108 candidate genes. In summary, these newly developed and integrated public SSR markers are an important genomic resource for both accelerating genetic analyses of complex traits and molecular breeding applications in peanut.

## Methods

### Plant materials and DNA isolation

A cultivated peanut that was de novo sequenced in our previous study [25], *A. hypogaea* cv. Fuhuasheng, was used as the reference genome for SSR identification. Two peanut varieties, Fuhuasheng and Yueyouhei4hao, were used for amplification and validation of the newly developed SSR markers by polymerase chain reaction (PCR). All of the materials were planted in a field in the summer season (July to November) at the experimental station of Guangdong Academy of Agricultural Sciences, Guangzhou, China. High-quality genomic DNA was extracted from young leaves using a Plant Genomic DNA Extraction Kit (BioTeke Corporation, Beijing, China) according to the manufacturer’s handbook (http://www.bioteke.com/en/?c=show&m=view&id=99).

### Identification of SSRs and design for SSR markers

The reference genome sequence of *A. hypogaea* cv. Fuhuasheng was downloaded from GenBank under accession number SDMP00000000 [25]. Perl scripts from MISA were used to perform SSR identification with the default parameters (http://pgrc.ipk-gatersleben.de/misa/). The identification criteria were as follows: mono-nucleotide repeat motifs with at least 12 repeats, di-nucleotide repeat motifs with five repeats, tri-nucleotide repeat motifs with four repeats, tetra-nucleotide repeat motifs with three repeats, and penta- and hexa-nucleotide repeat motifs with two repeats. Compound SSRs were defined as those with a < 100-nt interval between two repeat motifs [24, 38].

The forward and reverse primers of each SSR were designed based on the flanking sequences of SSR repeat motifs using Primer 3 [39]. Two Perl scripts, p3_in.pl and p3_out.pl, were used for the programmer-to-programmer data interchange between MISA and Primer 3.0 (http://pgrc.ipk-gatersleben.de/misa/primer3.html). The primer design criteria were as follows: primer length was between 18 and 27 bp, melting temperature was 57 to 63 °C, GC content ranged from 30 to 70%, and product size was from 100 to 300 bp.

An R package (http://www.R-project.org), Cmplot, was used to draw the high-density physical map of the newly developed SSR markers, and another R package, Rcircos, was used to visualize various features of each chromosome, such as gene position and distribution, the density of all SSR markers, and the distribution of the tested SSR markers.

### *In silico* evaluation and PCR validation of SSR markers

Four whole genome sequences, including those of two cultivated peanut landraces, *A. hypogaea* cv. Shitouqi [26] and Tifrunner [27], and their two diploid progenitors, *A. duranensis* and *A. ipaensis* [20], were used as templates for in silico evaluation of the designed SSR markers using electronic PCR (e-PCR) with the following parameters: 4-bp mismatch, 1-bp gap, and 0–2000-bp product size [40]. The paired primers should meet the following criteria: (1) only one in silico PCR product generated from any one template; (2) the SSR basic motif was as expected; and (3) the PCR product length was also as expected.

A total of 188 newly developed SSR primer pairs were arbitrarily selected and then synthesized for validation by PCR amplification. The PCR mixture was prepared in a 10-μl volume that contained 1 μl template DNA (~ 100 ng), 0.4 μl of each primer, 3.2 μl ddH_2_O, and 5 μl 2× Power Taq PCR Master Mix (BioTeke Corporation, Beijing, China). The PCR amplification program was as follows: (1) 95 °C for 5 min; (2) total of 35 cycles, each cycle was as below: 30 s at 95 °C, 30s at 55 °C for annealing, 30 s at 72 °C for extension; (3)10 min at 72 °C, and then storage at 4 °C. PCR products were separated and tested in Fragment Analyzer™ Automated CE System (Advanced Analytical Technologies, Inc., Beijing, China), and PROSize 2.0 was used to analyze and visualize the data (https://www.aati-us.com/support/software/).

### Integrating new and previously published SSR markers

In a previous study, we constructed an integrated consensus genetic map with published markers, including EST-SSR, transcriptome-SSR, and g-SSR markers [16]. Based on the forward and reverse primer sequences of each marker, these public SSR markers were mapped to the *A. hypogaea* cv. Fuhuasheng reference genome for integration with the newly developed SSR markers using e-PCR software with default parameters [40].

## Supplementary information

**Additional file 1:** **Table S1.** Number of SSRs and SSR markers identified on different chromosomes. **Table S2.** Summary of SSR motifs and repeats.

**Additional file 2:** **Figure S1.** Chromosome-wide distribution of SSRs in *A. hypogaea* cv. Fuhuasheng genome. **Figure S2.** Number of SSR repeat motifs. **Figure S3.** Abundance of the top 30 different types of SSR motifs. **Figure S4.** Number (A) and percentage (B) of different types of SSR markers. **Figure S5.** Summary of SSR types of the developed SSR markers. **Figure S6.** Product size of 188 SSR markers tested by PCR amplification. **Figure S7.** Number of loci in the public SSR markers as determined by e-PCR remapping. **Figure S8.** Comparison of known QTLs in genetic and physical maps.

**Additional file 3:** **Table S3.** Summary of SSR markers in subgenome A01–A05.

**Additional file 4:** **Table S4.** Summary of SSR markers in subgenome A06–A10.

**Additional file 5:** **Table S5.** Summary of SSR markers in subgenome B01–B05.

**Additional file 6:** **Table S6.** Summary of SSR markers in subgenome B06–B10.

**Additional file 7:** **Table S7.** Summary of SSR markers on nine scaffolds. **Table S8.** Summary of common SSR markers in four *Arachis* species. **Table S9.** Summary of 188 SSR markers tested by PCR amplification. **Table S10.** Summary of the public SSR markers with only a single locus match to the reference genome. **Table S11.** Candidate gene identification and annotation for the target QTL, *qLLS_T13_A05_7*.

## Data Availability

The datasets supporting the conclusions of this article are included within the article and its additional files.

## Abbreviations

ESTs: Expressed sequence tags
PCR: Polymerase chain reaction
QTL: Quantitative trait locus
SSR: Simple sequence repeats
