# Supplementary material for: Genome-wide identification of microsatellite markers from cultivated peanut (Arachis hypogaea L.)
Source: BMC Genomics. 2019 Nov 1;20:799. doi: 10.1186/s12864-019-6148-5 (PMC6824139; doi:10.1186/s12864-019-6148-5)
Supplement: Supplementary file 2 — Additional file 2: Figure S1. Chromosome-wide distribution of SSRs in A. hypogaea cv. Fuhuasheng genome. Figure S2. Number of SSR repeat motifs. Figure S3. Abundance of the top 30 different types of SSR motifs. Figure S4. Number (A) and percentage (B) of different types of SSR markers. Figure S5. Summary of SSR types of the developed SSR markers. Figure S6. Product size of 188 SSR markers tested by PCR amplification. Figure S7. Number of loci in the public SSR markers as determined by e-PCR remapping. Figure S8. Comparison of known QTLs in genetic and physical maps. [file 12864_2019_6148_MOESM2_ESM.pdf]

Genome-wide identification of microsatellite markers from cultivated peanut (*Arachis hypogaea* L.)

Supplementary Figures

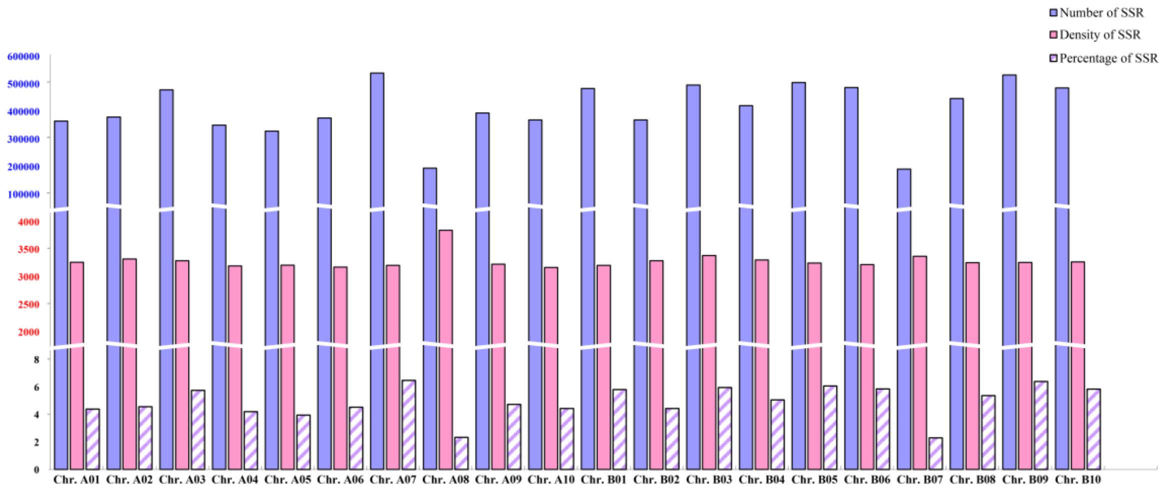

Figure S1 Chromosome-wide distribution of SSRs in *A. hypogaea* cv. Fuhuasheng genome.

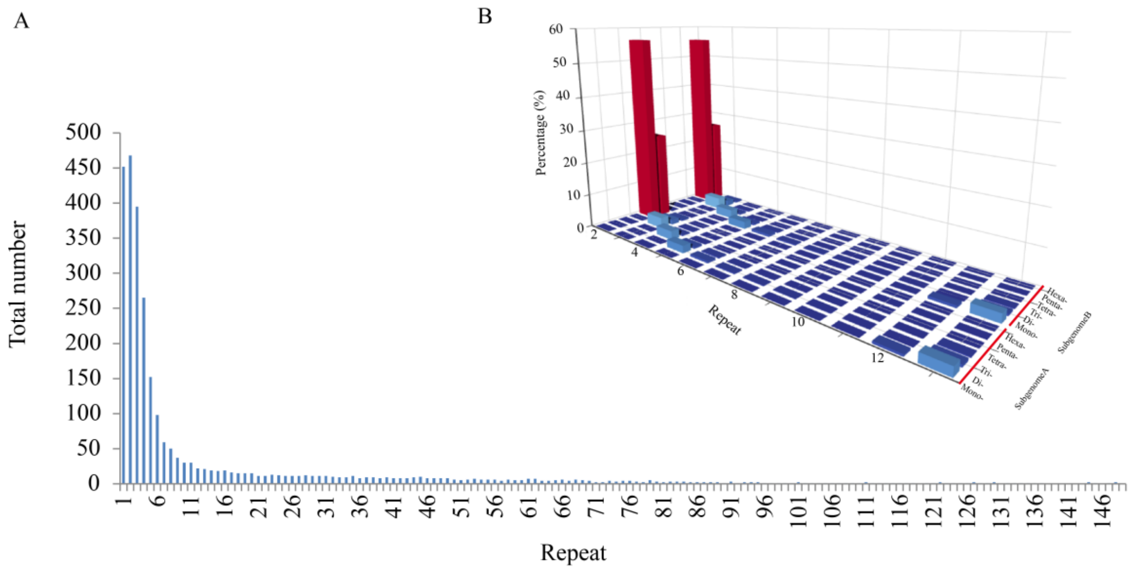

Figure S2 Number of SSR motif repeats. **a** Distribution of all SSR motif repeat number; **b** Distribution of SSR motif repeat number from mono- to hexa-nucleotide in subgenome A and B.

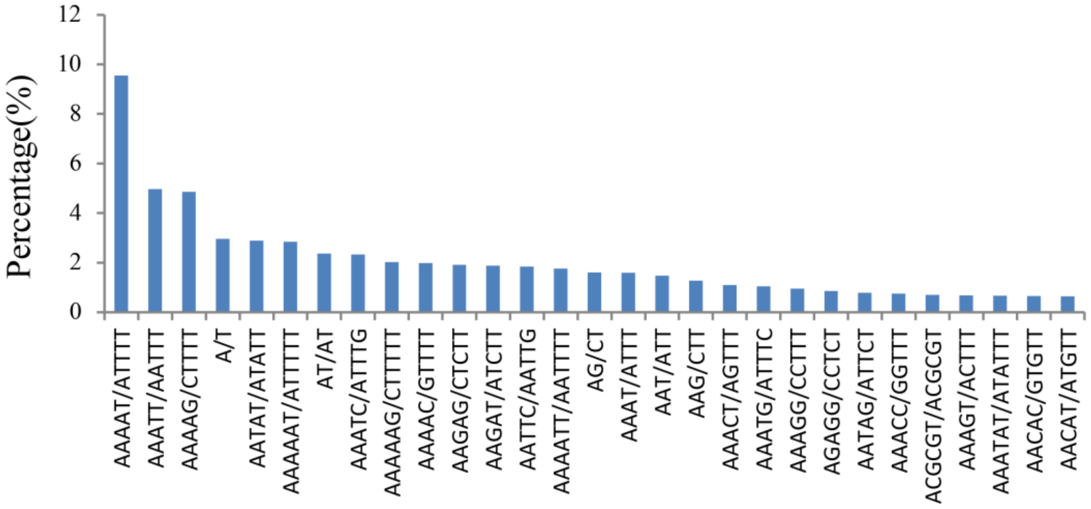

Figure S3 The abundance of the top 30 different types of SSR motifs.

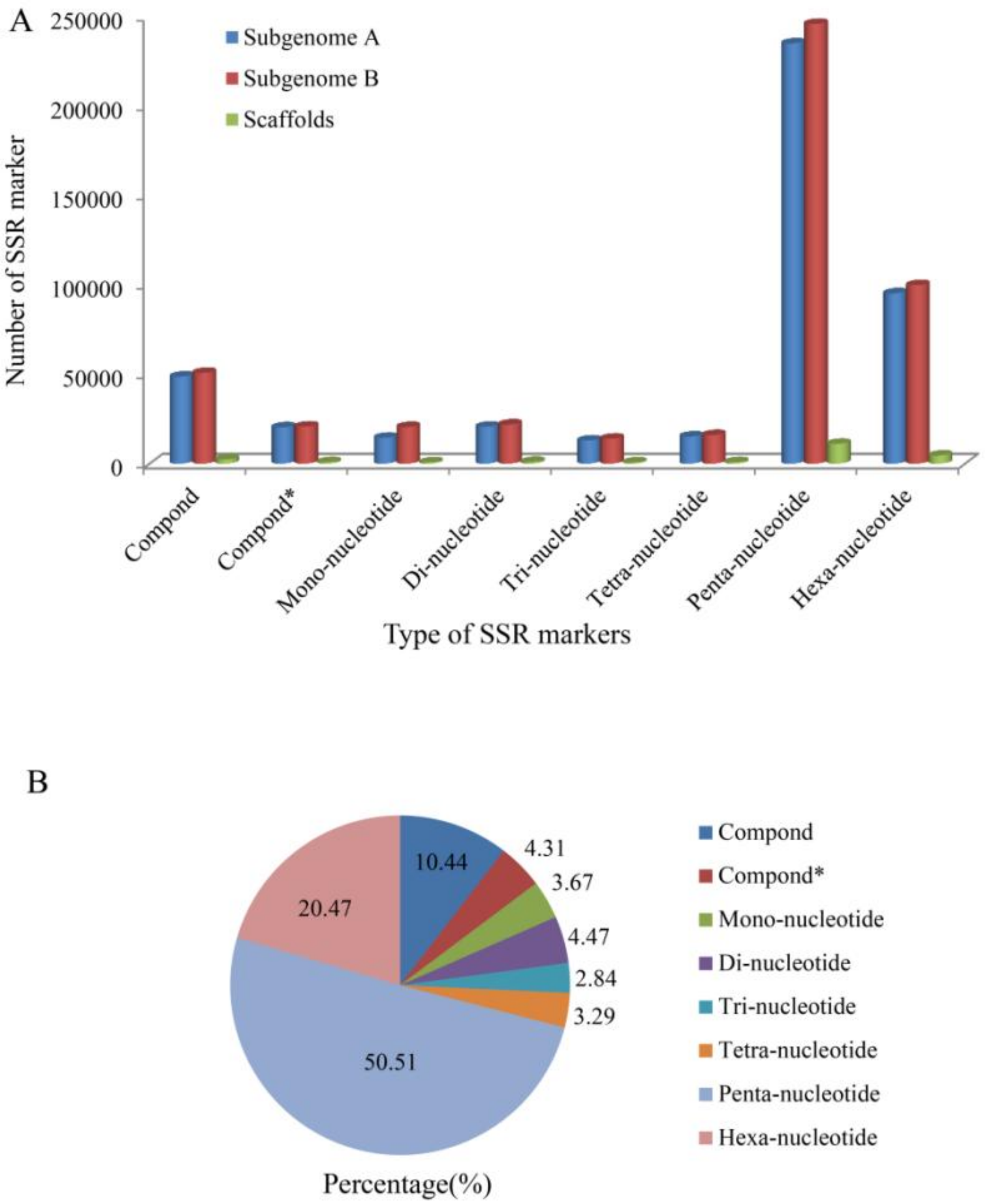

Figure S4 Number (A) and percentage (B) of different types of SSR markers.

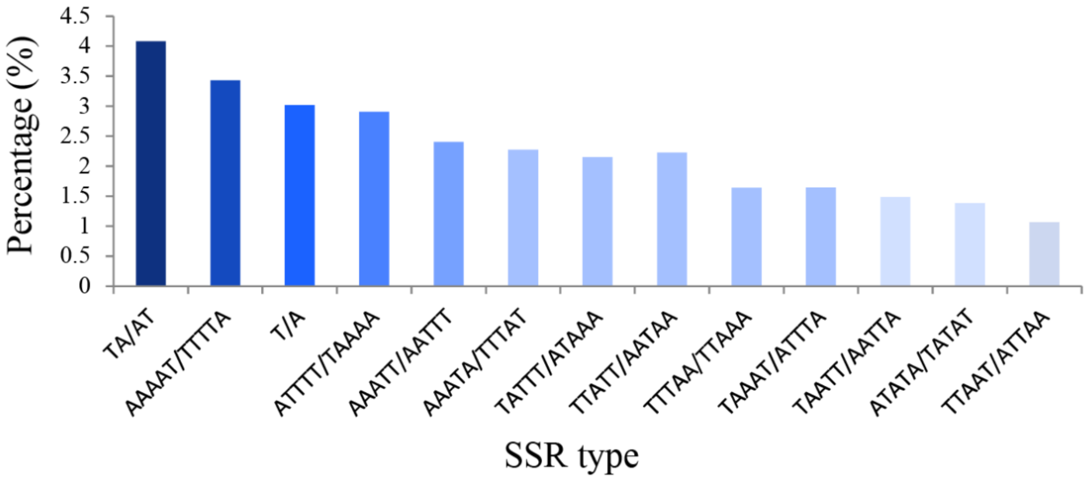

Figure S5 Summary of SSR types of the developed SSR markers.

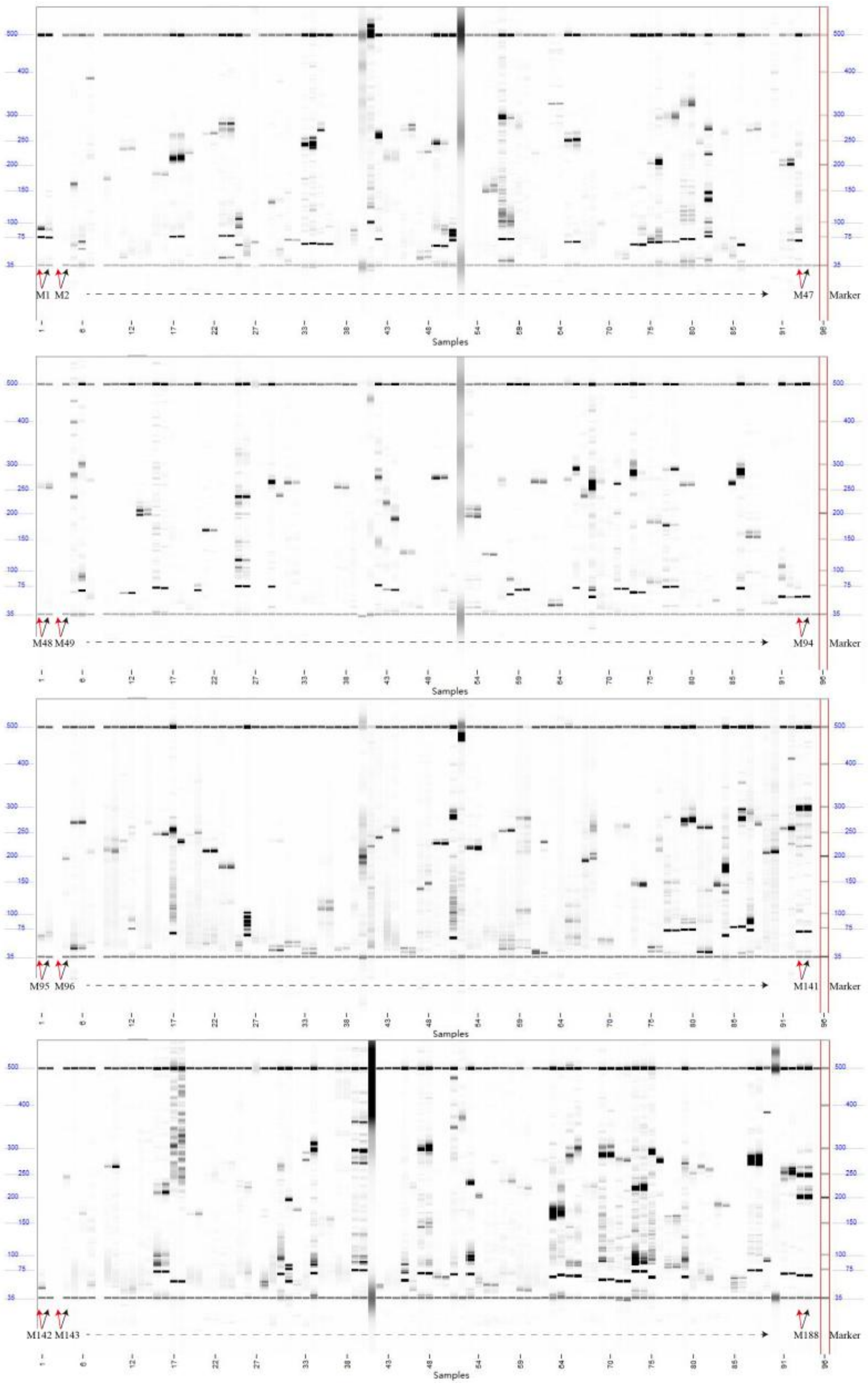

Figure S6 Product size of 188 tested SSR markers by PCR amplification. Red and black arrow represent Fuhuasheng and Yueyouhei4hao DNA templates, respectively.

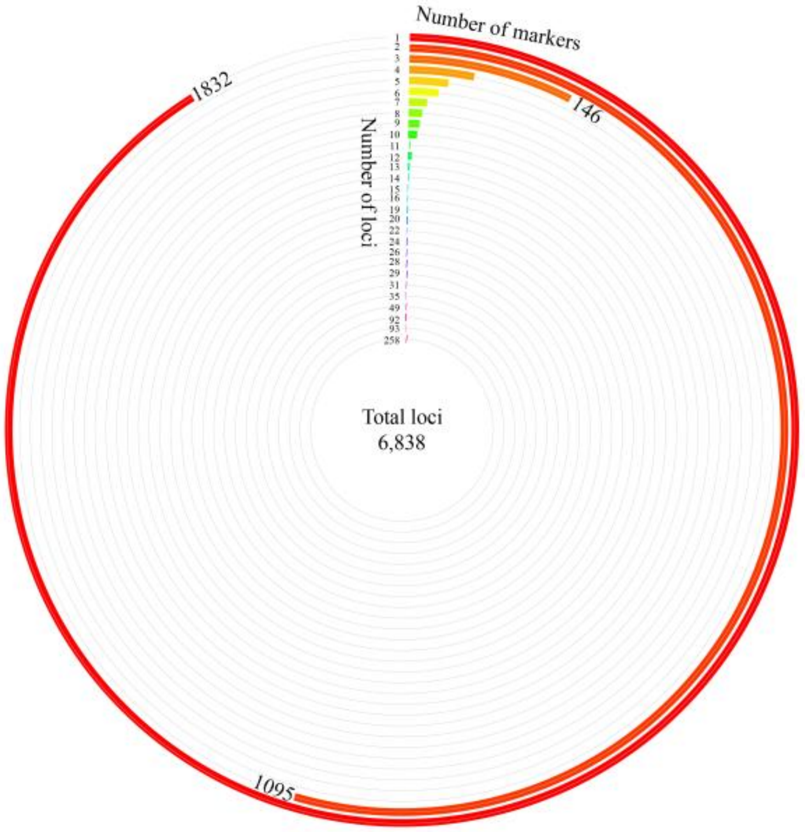

Figure S7 Number of loci for the public SSR markers as determined by e-PCR remapping.

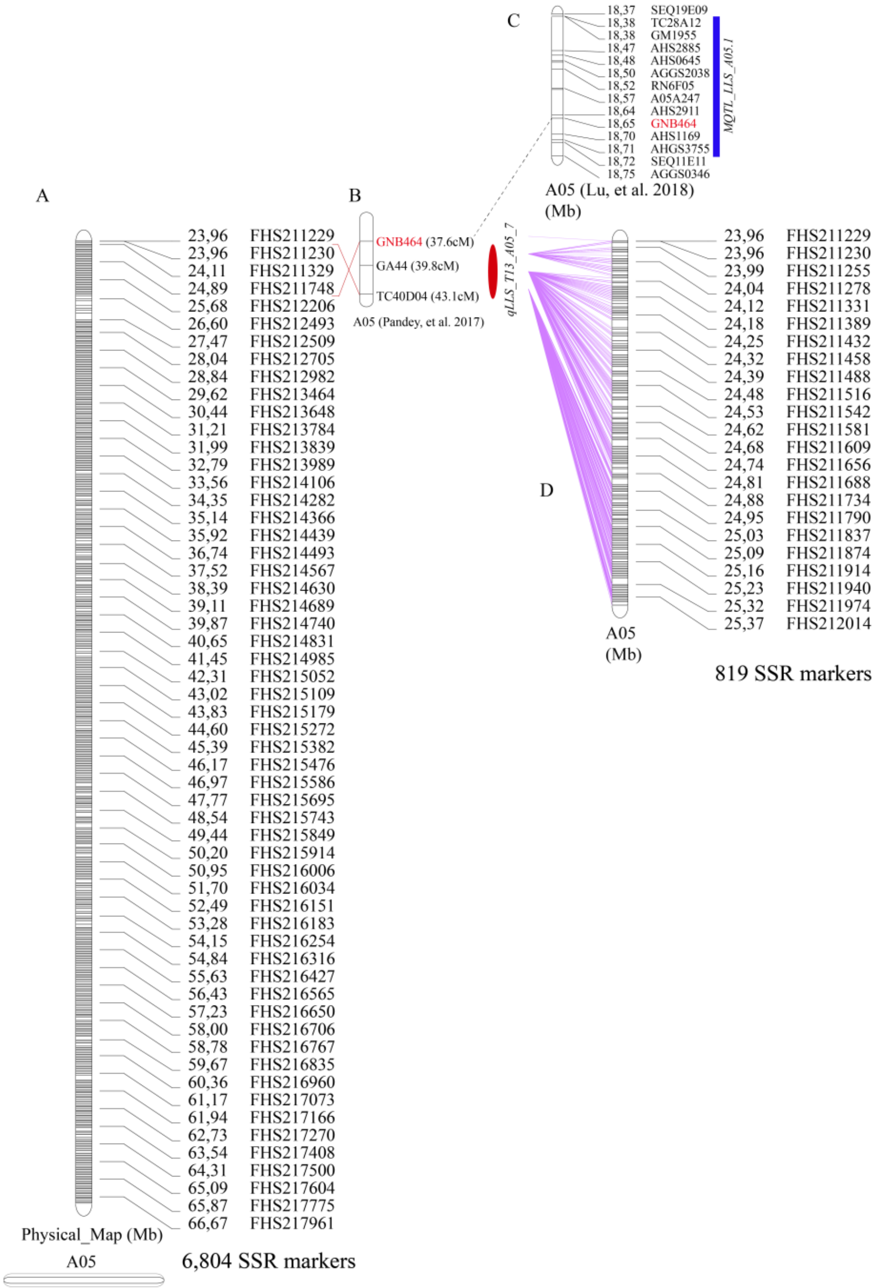

Figure S8 Comparison of known QTLs in genetic and physical maps. **a** A total of 6804 newly SSR markers were developed in target region of A05 physical map; **b** The genetic position of a late leaf spot QTL, *qLLS_T13_A05_7*, previously identified by Pandey, et al.; **c** The genetic position of a meta-QTL for late leaf spot, *MQTL_LLS_A05.1*, previously detected by our previous study (Lu, et al.); **d** A total of 819 newly available SSR markers in the target QTL region.

References

[1] Pandey MK, et al. Genetic Dissection of Novel QTLs for Resistance to Leaf Spots and Tomato Spotted Wilt Virus in Peanut (*Arachis hypogaea* L.). Front Plant Sci, 2017, 8:25.

[2] Lu Q, et al. Consensus map integration and QTL meta analysis narrowed a locus for yield traits to 0.7 cM and refined a region for late leaf spot resistance traits to 0.38 cM on linkage group A05 in peanut (*Arachis hypogaea* L.). BMC Genomics, 2018, 19:887.
